# Virus-Like Particles and Nanoparticles for Vaccine Development against HCMV

**DOI:** 10.3390/v12010035

**Published:** 2019-12-28

**Authors:** Michela Perotti, Laurent Perez

**Affiliations:** 1 Faculty of Biomedical Sciences, Institute for Research in Biomedicine, Università della Svizzera Italiana, 6500 Bellinzona, Switzerland; michela.perotti@irb.usi.ch; 2 Institute of Microbiology, ETH Zürich, 8093 Zürich, Switzerland; 3 European Virus Bioinformatics Center, 07743 Jena, Germany

**Keywords:** HCMV, cytomegalovirus, VLP, nanoparticle, vaccine, immune response

## Abstract

Human cytomegalovirus (HCMV) infects more than 70% of the human population worldwide. HCMV is responsible for high morbidity and mortality in immunocompromised patients and remains the leading viral cause of congenital birth defects. Despite considerable efforts in vaccine and therapeutic development, HCMV infection still represents an unmet clinical need and a life-threatening disease in immunocompromised individuals and newborns. Immune repertoire interrogation of HCMV seropositive patients allowed the identification of several potential antigens for vaccine design. However, recent HCMV vaccine clinical trials did not lead to a satisfactory outcome in term of efficacy. Therefore, combining antigens with orthogonal technologies to further increase the induction of neutralizing antibodies could improve the likelihood of a vaccine to reach protective efficacy in humans. Indeed, presentation of multiple copies of an antigen in a repetitive array is known to drive a more robust humoral immune response than its soluble counterpart. Virus-like particles (VLPs) and nanoparticles (NPs) are powerful platforms for multivalent antigen presentation. Several self-assembling proteins have been successfully used as scaffolds to present complex glycoprotein antigens on their surface. In this review, we describe some key aspects of the immune response to HCMV and discuss the scaffolds that were successfully used to increase vaccine efficacy against viruses with unmet medical need.

## 1. Introduction

Human cytomegalovirus (HCMV) is a ubiquitously distributed member of the *Herpesviridae* family belonging to the *Betaherpesvirinae* sub-family; it infects 70% of the human adult population worldwide [1,2] and primary infection is usually asymptomatic in immunocompetent individuals. Nevertheless, the virus establishes a lifelong latency of the host after a primary infection and viral reactivation is common, also it does not necessarily imply clinical symptoms [1,3]. However, infection or viral reactivation in immunodeficient individuals such as AIDS patients, solid organ (SOT) or hematopoietic stem cells (HSCT) transplant patients causes morbidity and mortality [4,5]. Furthermore, HCMV is the most common viral cause of congenital birth defects affecting 0.7% of the newborns with permanent sequelae such as sensorineural hearing loss, growth restriction, and cognitive disabilities [6]. Current antiviral therapies and transfusion with hyper-immune globulins to control viremia are not efficient [7]. Therefore, given the severity and importance of these diseases, as well as the associated socioeconomic cost, the need for an HCMV vaccine has been assigned in the highest priority category by the Institute of Medicine and it represents the second highest priority target after HIV by the Centers for Disease Control [8,9]. Over the last decades, considerable efforts were deployed to develop a vaccine capable of preventing HCMV infection, congenital transmission and viral spreading after SOT or HSCT from seropositive donors to seronegative recipients [10]. The potential vaccine included live virus, attenuated or vectored viral vaccines expressing HCMV immunogens as well as purified recombinant protein vaccines that have been evaluated in clinical trials [11,12]. The abundant virion envelope glycoprotein B (gB) was shown to elicit vigorous T cell and antibody responses and represents the basis of most vaccines developed so far [13]. In a recent phase II clinical trial the recombinant gB vaccine formulated in MF59 adjuvant (gB/MF59), an oil-in-water emulsion, generated antibody titers comparable to natural infection. However, the vaccine demonstrated only modest efficacy in preventing primary HCMV infections in seronegative women and in the reduction of viremia in transplant recipients [14,15]. Surprisingly, a recent study demonstrated that seronegative patients vaccinated with the gB/MF59 vaccine developed a faster humoral response against gB after solid organ transplantation from seropositive donors [16]. Still, it is of the utmost importance to evaluate possible strategies for HCMV next generation vaccines. In this review, we will summarize the current findings on the adaptive immune response to HCMV and provide an update on the new methodologies available to boost the immune response against infectious diseases using virus-like particles (VLPs) and nanoparticles (NPs).

## 2. Immune Response against HCMV

The establishment of a long-lasting immunity in response to a primary HCMV infection is required to control subsequent HCMV reactivation and prevent uncontrolled viral replication or serious HCMV diseases [17,18]. HCMV infection activates both humoral and cellular immune responses. It is commonly believed that cellular immunity controls most of HCMV replication. Nonetheless, HCMV-specific antibodies have been associated with the prevention and protection from reinfection as well as the congenital transmission of HCMV [6,19,20]. Despite this, it is clear that the humoral response on its own is not able to clear the virus and prevent viral reactivation [21,22]. Nevertheless, the two arms of the adaptive immune response appear to be necessary for protection and resolution of HCMV infection [23].

### 2.1. Cellular Immune Response against HCMV

The primary cellular response to HCMV infection arises from natural killer (NK) cells, which trigger the release of inflammatory cytokines and cause lysis or apoptosis of infected cells [24]. Secondly, T cells restrict HCMV viral replication and viral spreading, although they are not able to definitively clear the virus [25,26,27]. HCMV-specific T cells represent 10% of both the CD4^+^ and CD8^+^ memory compartments in the peripheral blood of CMV-seropositive individuals [28]. CD8^+^ T cells release perforin and granzyme B to promote lysis of infected cells that present CMV peptides on MHC-I [29,30], while CD4^+^ specific T cells present a typical Th1 cytokine secretion signature (IFN-γ and TNF-α) [31]. Immune repertoire interrogation using 13,687 spanning peptides to cover 213 predicted CMV-proteins [32] revealed that human T cells recognize at least 151 HCMV proteins. Among these, CD4^+^ T cells recognize 125 proteins, while CD8^+^ T cells recognize 107 proteins, and 81 proteins are recognized by both cell types. Although the HCMV-specific T cell response is very broad, the vast majority of the results points toward antigens that are highly conserved among different HCMV strains, such as the 65 kDa phosphoprotein (pp65) and the immediate early 1 (IE1) proteins, which are recognized by more than 50% of seropositive individuals [28]. CD8^+^ T cells were proven to have a protective role in both HSCT and SOT recipients [33,34,35] and a function in preventing congenital HCMV infection [36]. However, one of the paradoxes of the cellular immunity against HCMV comes from the observation that HCMV does induce a very large T cell response, meanwhile the virus also possesses sophisticated and numerous immune evasion mechanisms [37]. Among those, the capacity of the virus to enter a latency state has been the subject of intense investigation. Indeed, while primary infection of immunocompetent individuals by HCMV is rarely the source of disease, the opposite is true for individuals with an immune system that is still immature (fetus) or compromised (HIV/AIDS and transplantations). Consequently, several studies sought to identify the antigens that are relevant for the latency phase. A vaccine able to boost the immune system against the latter would help clearing the latent reservoir of the virus. It is known that HCMV establishes latency in the myeloid cell lineage and viral genome has been detected in peripheral blood monocytes and in CD34^+^ progenitors in the bone marrow [38,39,40]. The viral gene expression program in these cells is restricted and extremely different from the one established during lytic infections. HCMV latent transcriptome is an area of extensive research, and at present, the exact roles of many latency-associated genes remain unclear. Nevertheless, several studies identified a number of viral genes that are specifically associated with latency [41,42]. Notably, RNAs from the major IE region (UL122–123 CLTs) [43], Latency Unique Natural Antigen (LUNA) [44], UL138 [41], UL111a (encoding a viral IL-10 homologue (LAcmvIL-10)) [45], UL144 [46] and US28 [47] were identified [22]. Moreover, viral reactivation is also associated with the expression of the major immediate early proteins (IE72 and IE86) [48]. T-cell repertoire interrogation identified four viral proteins expressed during latency (LUNA, UL138, US28, and LAcmvIL-10). Interestingly, these proteins can be recognized by CD4^+^ T cells and T-cell responses specific for all four proteins are detectable in healthy HCMV-positive donors [49]. Accordingly, future vaccine candidates should boost cellular immunity against some of these proteins representing key targets for viral clearance.

### 2.2. Humoral Immune Response against HCMV

Upon HCMV infection, a large quantity of the antibodies generated target the most abundant viral proteins. These include proteins from the tegument (i.e., pp65), the immediate early proteins (i.e., IE1), and envelope glycoproteins such as gB, gM/gN, and the gH/gL complexes (trimer and pentamer). Antibodies targeting antigens that are not exposed on the surface of the virion are unlikely to generate an efficient protection against the virus [10]. Nonetheless, antibodies targeting the envelope glycoproteins are able to neutralize viral infection in vitro and correlate with a decrease of viral transmission in primary infected pregnant women [50]. It is known that antibodies targeting the pentamer are the most potent neutralizers at least in vitro [51,52], while antibodies targeting gHgL and gB are a thousand times less potent [51]. This result is still not fully understood, since the gHgL and gB proteins form the core machinery for viral membrane fusion [53] and monoclonal antibodies (mAbs) targeting the fusion machinery of other herpesviruses, such as Epstein–Barr virus, are potent neutralizers [54]. Immune repertoire interrogation of HCMV seropositive donors allowed the identification of multiple mAbs targeting the pULs subunits. We recently demonstrated that these mAbs neutralize the virus by blocking the molecular recognition of the pentamer to its cellular host receptor Neuropilin 2 [55]. In contrast, mAbs targeting gH are supposed to prevent the activation of the fusion machinery [56]. These mAbs were assigned to only two antigenic regions of gH, indeed 3G16 and MSL-109 mAbs bind toward the C-terminus of gH, while the 13H11 mAb binds close to the gL interaction site [57].

Recently, two studies reported the results of the recombinant gB vaccine formulated in MF59 adjuvant, which demonstrated partial efficacy in reducing viraemia after SOT and preventing primary infection in women and adolescents [14,15]. Sera analysis from gB-vaccinated individuals allowed the characterization of at least five gB antigenic domains (AD-1 to AD-5). The AD-1 is a domain of 80 amino acids between amino acid 560 and 640 whereas AD-2 consists of two discontinuous binding sites: Site I, located between amino acids 68 and 77, is highly conserved among strains and is considered as a target of neutralizing responses, while Site II, located between amino acids 50 and 54, is less conserved. AD-3 is a linear epitope corresponding to amino acids 798–805 in the intraluminal region of gB. AD-4, like AD-2, is a discontinuous domain corresponding to amino acids 121–132 and 344–438. AD-5 matches to amino acids between 133 and 343 [58,59,60]. Interestingly, Baraniak et. al. demonstrated that vaccination only boosted AD-2 responses in the 50% of CMV+ individuals with a preexisting response and did not induce a new AD-2 response in those who lacked AD-2 antibodies following natural infection [15,61]. In parallel, Nelson et al. observed an increase of anti-gB IgG titer induced by the same vaccine in a cohort of CMV-post-partum women [14]. However, the authors also subsequently observed that 76% of the vaccine-induced IgG response recognized AD-3. Considering that AD-3 is located in the intraluminal part of the virus, antibodies against this region will be most probably non-protective [12]. The hypothesis that AD-3 is diverting the immune response away from antigenic sites that more likely induce protective antibody responses (AD-2), was recently proposed [62]. The next HCMV vaccines will have to contain multiple antigens that should induce a strong neutralizing antibody response protecting from primary infections and an efficient T-cell response to prevent viral reactivation and to clear the latent virus.

## 3. HCMV Antigens as Vaccine Candidates

**Figure 1:**
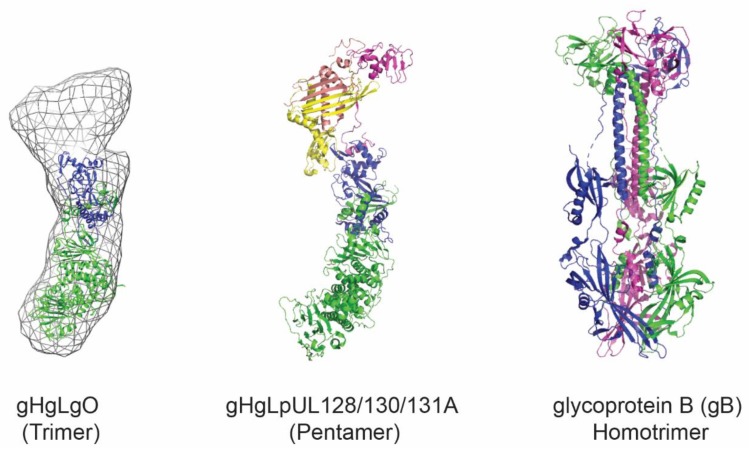
Structural representation of HCMV glycoproteins. Shown is the trimer, composed of the gHgL heterodimer and gO. Representation prepared with PDB: 5voc and EMD-3391. The pentamer is composed of the gHgL heterodimer and the three additional subunits pUL128, pUL130, and pUL131A. Representation prepared with PDB: 5voc. The gB homotrimer in its postfusion conformation is shown based on PDB: 5cxf. The figure was prepared using PyMOL software (The PyMOL Molecular Graphics System, Version 4.5 Schrödinger, LLC).

HCMV is the largest virus infecting humans, with a genome containing a linear double-stranded DNA of about 230 kb, from which at least 170 open reading frames (ORFs) have been identified [63,64]. The virus contains over 30 structural proteins and encodes at least 20 membrane glycoproteins expressed on the virion envelope, which are involved in immune evasion, cell attachment, and viral entry. HCMV exhibits an extremely broad cellular tropism being able to infect different cell types ranging from epithelial, endothelial, fibroblasts, myeloid hematopoietic precursors, monocytes/macrophages to smooth muscle cells, stromal cells, neurons, astrocytes, hepatocytes and glial cells [65]. Identification of the host cellular receptors has been a subject of debate during the last decade. It is now accepted that the two main host cell receptors for HCMV involve the platelet-derived growth factor receptor α (PDGFRα) [66,67,68] and Neuropilin 2 (Nrp2) [55]. In addition, other cellular host factors were described as facilitators of viral entry such as the olfactory receptor family member OR14I1 [69], the adipocyte plasma membrane-associated protein (APMAP) [70], CD46 [71], and CD147 [72]. However, the biological relevance of this multitude of receptors remains to be understood. In contrast, the viral ligands responsible for the broad cellular tropism were clearly identified and correspond to two viral envelope complexes: the gHgLgO (trimer) and the gHgLpUL128pUL130pUL131A (pentamer) (Figure 1) [73]. The trimer is a heterocomplex, in which the heterodimer consisting of gH (UL75) and gL (UL115) is disulfide-linked to glycoprotein O (gO), a heavily N-glycosylated polypeptide encoded by UL74 [74,75,76]. The trimer complex binds PDGFRα and is required for entry in all cell types [77], representing an attractive target for vaccine design. However, the gO sequence heterogeneity and the large glycan shield present on this subunit are impairing the generation of potent neutralizing mAbs. Along with others, we speculate that these issues will probably slow down possible efforts to use the trimer as a vaccine candidate [78]. The pentamer complex is composed of the gHgL heterodimer bound to three additional glycoproteins encoded by pUL128, pUL130, and pUL131A. The pentamer complex is required for viral entry in epithelial, endothelial, and myeloid cells and binds Nrp2 [55,65,79]. It represents the main target of HCMV neutralizing antibodies, eliciting a protective response targeting the pULs subunits, which is several orders of magnitude more potent than any other response against HCMV glycoprotein [51,52,80]. However, anti-pULs mAbs are not able to block infection in all cell types, in contrast to mAbs directed against gH and gB subunits [81]. Indeed, membrane fusion and cell entry in all cell types is dictated by the core fusion machinery composed of gHgL and the glycoprotein B (gB) which are thus considered as primary targets for vaccine development [2]. gB is a class III viral fusion protein that forms homotrimers (Figure 1) and was initially reported to bind cell–surface proteins such as PDGFRα and EGFR. However, it is more likely that gB functions as a viral fusogen that is triggered when viral and host membranes are in close proximity [82]. In addition to the HCMV membrane glycoproteins, other viral antigens have been considered as potential vaccine targets. The phosphoprotein 65 (pp65) encoded by pUL83 and the immediate-early protein 1 (IE1) encoded by pUL123, both eliciting a robust T cell response, have undergone evaluation as subunit or vectored vaccines in human [83,84]. In conclusion, the aforementioned vaccine candidates failed to confer an appropriate protection from infection, viral reinfection or reactivation. We believe that the development of a protective vaccine should include antigens from the viral envelope to trigger the humoral response (mainly pentamer and newly designed gB) and antigens from the tegument or proteins that are specifically associated with latency (i.e., US28) to prompt the cellular response able to control viral reactivation.

## 4. Virus-Like Particles and Nanoparticles for Antigen Display

Virus-like particles (VLPs) and nanoparticles (NPs) are protein structures that resemble wild type viruses but do not have a viral genome nor infectious ability, creating in principle safer vaccine candidates. Both VLPs and NPs are composed of self-assembling proteins displaying the epitope of interest at a high density on their surface. Antigen presentation as a repetitive array is known to drive stronger humoral immune responses when compared to the single soluble antigen [85]. This effect is thought to derive from some key properties of the VLPs/NPs presentation such as the increased antigen density that creates avidity leading to a stronger B cell activation through antigen-driven cross-linking of B cell receptors (BCRs) [86]. Moreover, VLPs/NPs are also supposed to enhance antigen uptake by antigen presenting cells (APCs) which subsequently support the adaptive arms of the immune system [87]. Finally, VLPs/NPs have also been proven, in some cases, to generate an epitope-focusing effect in which antigenic sites promoting the generation of potent neutralizing mAbs were preferentially targeted [88,89]. Over the last decades, several platforms for VLPs/NPs design have been generated, including the usage of viral core proteins, covalent links of individual folded proteins through site-specific ligations and de novo protein nanostructure formation via non-covalent intramolecular and intermolecular interactions. VLPs/NPs are currently recognized as one of the most promising and extensively studied molecular carriers for new vaccine development. Here we will present the current VLP HCMV vaccines in development and provide some examples that can be relevant for novel vaccine design.

### 4.1. Developed HCMV VLP Vaccine

Enveloped virus-like particles (eVLPs) are VLP structures wrapped with a lipid envelope obtained by transfection of the Moloney murine leukemia virus (MLV) gag protein in human or hamster cell lines (i.e., HEK or CHO cells). The MLV gag expression induces burgeoning of particles from membranes of transfected cells. Variation Biotechnologies Incorporated (VBI) laboratories generated an eVLP vaccine expressing an HCMV gB full-length or a chimeric gB protein, where the extracellular domain (ECD) of gB is membrane-anchored using the transmembrane and cytoplasmic domains of the vesicular stomatitis virus (VSV) G protein [90]. The chimeric gB-VSV-G protein is thought to maintain gB in a prefusion conformation. Both vaccines were used to immunize mice and induce a neutralizing antibody response 10-fold higher compared to their soluble recombinant protein counterpart [90]. The vaccine was entered in phase I clinical studies enrolling HCMV-seronegative individuals for evaluation of safety and immunogenicity in early 2016 [91]. An additional eVLP vaccine candidate, targeting both the gB and pp65 antigens, has also been manufactured by VBI. However, we believe that a combination of gB, pentamer, and pp65 or US28 would be more appealing. Finally, another eVLP HCMV vaccine candidate was developed by Redvax GmbH, a Swiss biopharmaceutical company. Unlike VBI, that used mammalian cells, they used the rePAX baculovirus co-expression technology minimizing the purification issues due to the relative large size of VLPs for the efficient generation of a VLP vaccine candidate containing HCMV pp65 and gB [92]. Redvax GmbH was later acquired by Pfizer and the vaccine was able to elicit high-titer neutralizing antibodies and good T cell responses after immunization. Nonetheless, no protection was demonstrated from viremia upon challenge.

### 4.2. Hepatitis B Core Antigen Nanoparticle

**Figure 2:**
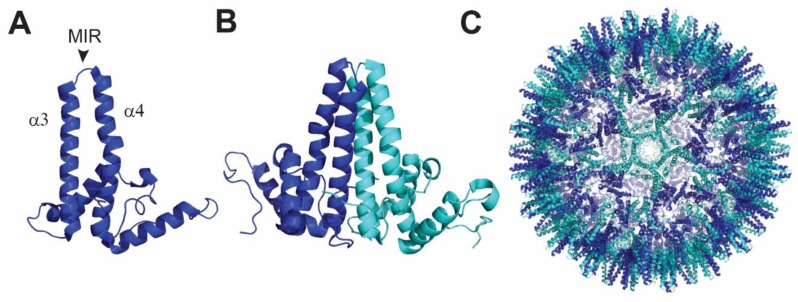
Hepatitis B core antigen and relative nanoparticle. (**A**) Polypeptide fold and structure of HBcAg monomer. The antigen of interest needs to be inserted in the MIR loop connecting alpha helices 3 and 4. (**B**) Two HBcAg monomers spontaneously interact to form a dimer, which acts as an intermediate in the nanoparticle formation. (**C**) Assembled nanoparticle containing either 90 or 120 dimers. The figure was prepared with PDB: 6htx using Pymol software (The PyMOL Molecular Graphics System, Version 4.5 Schrödinger, LLC).

The core protein of the hepatitis B virus (HBc) self-assembles into highly immunogenic virus-like structures with icosahedral symmetry. The hepatitis B core nanoparticle (HBc) is assembled from dimers of 183 or 185 amino acids and occurs in two size classes with a T = 3 or T = 4 symmetry. Depending on the symmetry, HBc is formed by 90 or 120 dimeric subunits with a respective diameter of 24 or 31 nm [93]. The antigen of interest needs to be inserted between alpha helices 3 and 4 of the major immune-dominant region (MIR) domain (amino acids 78–82) (Figure 2A) of the HBc protein. This insertion allows the antigen to be exposed on spike structures at the surface of the assembled nanoparticle. HBc protomers first dimerize and then multimerize (Figure 2B,C). Several developments led to the success of this platform, such as splitting of the HBc protein between the α3 and α4 helices or fusing two monomers together using a flexible glycine serine linker that allowed the introduction of larger protein domains [94,95]. However, one limitation of this technology is the need for both the N- and C-termini of the inserted antigen to fit with the geometry of the core acceptor site, a requirement that is not always successfully met by natively folded proteins.

In summary, the HBc is a powerful platform to generate nanoparticles and it is particularly useful in the case of small antigens. Indeed, it is unlikely that complex envelope glycoproteins such as gB or the gHgL complexes could be correctly displayed on HBc particles. Nevertheless, HBc could be very efficient to generate strong T cell responses [96]. For example, the viral-encoded G protein-coupled receptor US28 was recently shown to aid in the establishment and the maintenance of viral latency. US28 modulates host–cell proteins to suppress viral processes associated with lytic replication and thereby represent an important target promoting latent infection [97]. HBc particles displaying US28 peptides recognized by T cells could provide an important advancement to boost the existing immune responses necessary to control reactivation of latently infected individuals [98].

### 4.3. Ferritin-Based Nanoparticle

**Figure 3:**
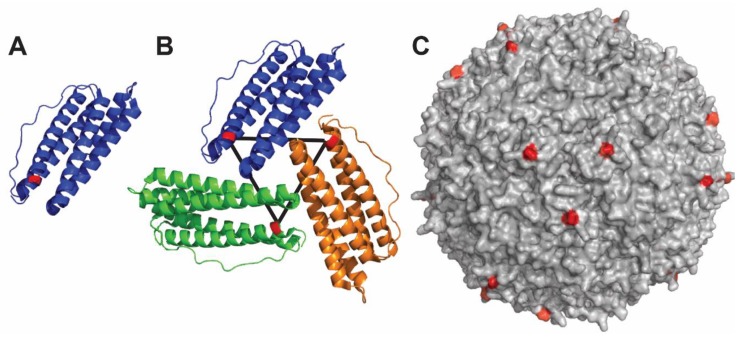
Structure of ferritin nanoparticle. (**A**) The antigen of interest is fused to the N-terminus of the ferritin polypeptide (shown in red). (**B**) Ferritin assembles to form symmetry units with threefold axes. (**C**) Fully assembled nanoparticle with surface-exposed N-termini colored in red. The figure was prepared using PDB: 3bve using PyMOL software (The PyMOL Molecular Graphics System, Version 4.5 Schrödinger, LLC).

Ferritin is an intracellular globular protein that stores iron and releases it in a controlled fashion [99]. The protein is expressed in many tissues, mainly as a cytosolic protein, but it can be also found at low concentration in the serum where it functions as an iron carrier [100]. Ferritin is a four-helix bundle protein consisting of two couples of anti-parallel α-helices connected by a long loop and a short C-terminal α-helix (Figure 3A). The monomer can dimerize and further self-assemble to form a nanocage of 24 protein subunits, which has an internal and external diameter of 8 and 12 nm, respectively [101]. Ferritin nanoparticle (ferritin-NP) presents the advantage of being resistant to thermal and chemical stress [102]. Moreover, antigen display is achieved through genetic fusion at the N-terminus of each ferritin protomer. Ferritin-NP self-arranges as an octahedron composed of eight trimeric units (Figure 3B), each with a 3-fold symmetry axis that allows the correct presentation of trimeric antigens at the nanoparticle surface (Figure 3C).

A recent development and one of the major advantages of this carrier is the possibility to generate multivalent displays of different antigenic proteins. For example, ferritin-NP was first used to present HIV-1 and influenza antigens on the same particle [103]. Furthermore, Kanekiyo and coworkers at the Vaccine Research Center in the United States. further developed and exploited the ferritin-NP system using a ferritin hybrid protein [89] to generate a universal vaccine against influenza. The team co-displayed the receptor binding domain (RBD) of the last 90 years H1N1 influenza viruses [104] and showed that immunization with this mosaic RBD-NP elicited a broad antibody response covering all the known H1N1 strains. Multiple displays on ferritin-NP have opened up new possibilities for novel vaccines against different pathogens or against different antigens of a given pathogen.

In the case of HCMV, we can speculate that a mosaic ferritin-NP displaying both pentamer and gB ECDs should generate a potent humoral immune reaction. The antibodies generated targeting the gHgL and gB subunits will block infection of connective tissues and stroma (containing fibroblasts). On the other hand, antibodies directed against the pUL subunits will block infection of epithelial tissues.

### 4.4. Qβ Nanoparticle

**Figure 4:**
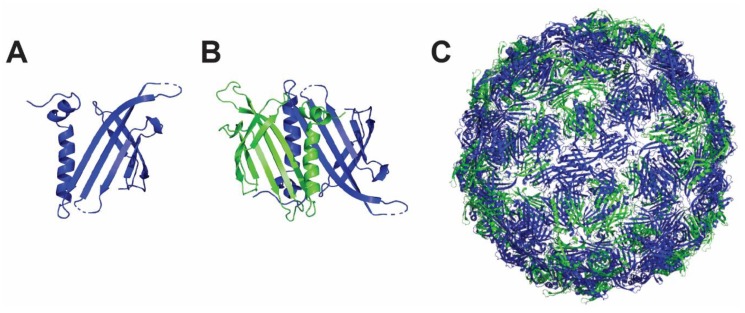
Structure and assembly of Qβ nanoparticle. Qβ protein is depicted as a monomer (**A**) and noncovalent dimer (**B**). (**C**) The fully self-assembled Qβ nanoparticle consists of 20 hexamers and 12 pentamers. The figure was prepared using 1qbe PDB structure and PyMOL software (The PyMOL Molecular Graphics System, Version 4.5 Schrödinger, LLC).

The bacteriophage Qβ is a member of the *Leviviridae* family, forming 30 nm icosahedral nanoparticles of 180 copies from the 14 kDa coat protein [105]. The coat protein monomer (Figure 4A) forms Qβ dimers (Figure 4B) that further assemble into pentamers and hexamers by disulfide and hydrogen bond interactions to form a Qβ nanoparticle under physiological conditions (Figure 4C). This nanoparticle is highly stable and is used to display ligands and proteins on its exterior surface [106,107]. Co-expression of Qβ proteins displaying different antigens of interest or engineered Qβ proteins displaying functionalized groups for subsequent direct antigen conjugation can be easily produced in bacterial cells. Of note, the genetic fusion occurs at the C-terminus of the protein, a property that might impair the correct folding of some antigens of interest. Genes encoding the viral Qβ protein have been also successfully expressed in yeast cells but the possibility to generate a Qβ nanoparticle in prokaryotic hosts, maintaining therefore a low production cost, will be of great interest for the generation of a personalized T cell vaccine targeting the proteins associated to latency. Regarding mammalian cell expression, there is currently no evidence that the system is also compatible with it, which is a prerequisite for the production of most of the HCMV glycoprotein antigens.

### 4.5. De Novo Design-Based Nanoparticle

**Figure 5:**
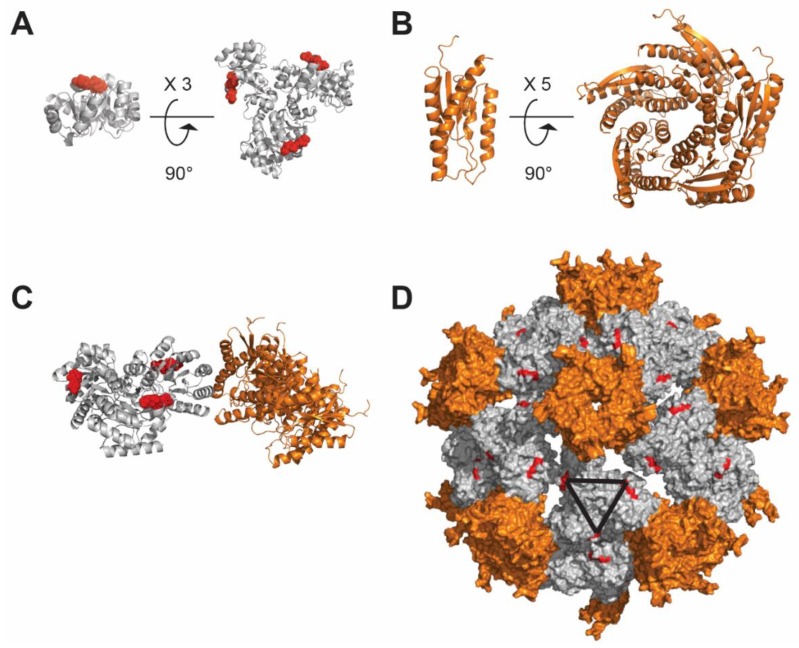
Self-assembly of the I5350 nanoparticle. (**A**) Polypeptide I53–50A.1NT1; shown in red is the N-terminus projecting outward, where antigens can be displayed. I53–50A.1NT1 naturally trimerizes forming a 3-fold symmetry axis. (**B**) The I53–50B.4PT1 protein (orange) assembles into a pentamer. (**C**) I53–50A.1NT1 trimer and I53–50B.4PT1 pentamer self-assemble as a nanoparticle. (**D**) Assembled nanoparticle formed by 20 trimers and 12 pentamers, the N-termini of I53–50A.1NT1 is shown in red, and the black triangle represents the 3-fold symmetry axis of each pair of trimers. The figure was prepared using PyMOL software (The PyMOL Molecular Graphics System, Version 4.5 Schrödinger, LLC).

Structure-based design of nanoparticle immunogens has been limited by the restricted number of scaffolds available and the fact that their physico-chemical properties are fixed (i.e., ferritin, encapsulins). Moreover, most of the self-assembling scaffolds spontaneously do so upon expression in the transfected host cell, a property that might lead to poor production yield. These constraints have pushed the exploration of new structural and functional geometries (i.e., icosahedron, dodecahedron) in the nanoparticle immunogen design field. Recent computational methods for designing novel self-assembling proteins with atomic-level accuracy offer the possibility to design self-assembling proteins with customized structures, offering new opportunities for structure-based vaccine design [108,109,110]. For instance, the HIV gp41 ectodomain trimer was displayed onto the I3–01 particle [108,111]. The gp41 antigen exposed on NPs, stimulated mAb-expressing B cells more effectively than the soluble trimers [111]. Two additional publications highlighted the potential of this de novo self-assembling nanoparticle for antigen display. The I5350 nanoparticle (Figure 5) was used to generate an enhanced vaccine against the respiratory syncytial virus (RSV). Self-assembling protein nanoparticles displaying 20 copies of the stabilized version of the RSV fusion glycoprotein trimer (DS-Cav1) induced a neutralizing antibody response ∼10-fold higher than soluble trimeric DS-Cav1 [112]. In the second case, native-like HIV-1 envelope trimer antigens were displayed in a multivalent fashion on the I5350 nanoparticle scaffold and immunization studies revealed a more effective priming compared to the soluble SOSIP trimers [113]. SOSIP corresponds to a stabilized trimer with mutations that cross-link the cleaved gp120 and gp41. In contrast, the natural HIV trimer is comprised of three copies of a non-covalently linked gp120/gp41 heterodimer arising from cleavage of the viral gp160 precursor protein. It can be speculated that de novo design-based nanoparticles will have an increasing impact on next generation vaccines since they are customizable at will, supported by a multitude of expression platforms and able to generate multivalent display as well as encapsulation of small proteins or compounds [114,115].

Ideally, I5350 particles should present on their surface the pentamer and gB to generate a humoral response against the gH, gL, pULs, and gB molecules. Moreover, as I5350 nanoparticles are hollow, the in vitro assembly step with I53–50B.4PT1 could be performed in the presence of T-cell stimulating peptides (i.e., pp65, IE1 and US28) that would be encapsulated inside the particles. The resulting vaccine candidates should in this way stimulate both arms of the immune system, offering a maximal protection.

### 4.6. SpyCatcher and SpyTag for Nanoparticle Formation

The SpyTag/SpyCatcher system (referred as Spy-System) is based on the internal isopeptide bond of the CnaB2 domain of FbaB protein from *Streptococcus pyogenes*. An internal isopeptide bond forms spontaneously in this domain between the reactive amine of lysine K31 and the side chain carboxyl of aspartate D117. This reaction is catalyzed by the spatially adjacent glutamate E77. The resulting isopeptide bond confers high stability to the CnaB2 domain. The CnaB2 domain is cleaved into two subcomponents, the large and incomplete immunoglobulin-like domain, named SpyCatcher, composed of 138 amino acids, and the short beta strand, named SpyTag, composed of 13 amino acids [116]. While the SpyCatcher contains the reactive lysine (K31) and catalytic glutamate (E77), the SpyTag includes the reactive aspartate (D117). The two components can still recognize each other with high affinity and the isopeptide is formed within minutes. Despite the fact that this system is not able to generate nanoparticles on its own, the two components of the Spy-System can be genetically fused to a number of viral or designed self-assembling proteins leading to the generation of a stable nanoparticle displaying a large variety of antigens. Moreover, the Spy-System offers a great flexibility since components can be inserted at the N- or C-termini of the target proteins. Its simplicity is of particular interest to quickly screen if a nanoparticle formulation will be of benefit for vaccine design, before generating genetic fusion on the nanoparticle of interest. The first nanoparticle assembly with Spy-System was performed by genetically fusing the SpyCatcher to the N-terminus of the viral coat protein (CP3) of the RNA bacteriophage AP205 [117]. AP205 VLPs have been then further investigated as vaccine candidates against a number of targets, including malaria and cancer [117,118,119].

## 5. Conclusions and Future Directions

We described a large variety of systems to generate both VLPs and NPs. Self-assembling scaffolds have been used to present complex glycoprotein antigens and for vaccination studies against influenza [104], HIV [113,120,121,122,123], Epstein–Barr virus [89], and RSV [112]. In all cases, antigen immunogenicity was increased by multivalent presentation and in some cases, an epitope focusing effect could be observed. The rise of computational design to generate novel self-assembling proteins has now paved the way for new possibilities to display antigens without limitation in term of quantity or oligomeric state of the antigen. HCMV is still the principal viral cause of congenital malformations. About one quarter of infants infected by HCMV in utero will develop severe sequelae including microcephaly, sensorineural hearing and vision loss, or cognitive delay [1,6]. The recent identification of key HCMV antigenic targets for both humoral and cellular immune responses and the capacity to display and encapsulate proteins or nucleic acids (RNAs) on VLPs/NPs is likely to generate improved vaccine candidates able to redirect the immune response against specific targets.
